# Effects of microRNA-208a on inflammation and oxidative stress in ketamine-induced cardiotoxicity through Notch/NF-κB signal pathways by CHD9

**DOI:** 10.1042/BSR20182381

**Published:** 2019-05-17

**Authors:** Hongjie Yuan, Shibin Du, Youliang Deng, Xiaoqing Xu, Qian Zhang, Miao Wang, Ping Wang, Yi Su, Xiao Liang, Yanyan Sun, Zhengzhuang An

**Affiliations:** 1 Department of Pain Medicine, Nantong Hospital of Traditional Chinese Medicine, Nantong 226001, China; 2 Department of Pain Medicine, Affiliated Hospital of Shaanxi University of Traditional Chinese Medicine, Xianyang 712000, China; 3 Department of Anesthesiology, Shenzhen University General Hospital, Shenzhen University Clinical Medical Academy, Shenzhen University, Shenzhen 518055, China; 4 Department of Anesthesiology, Xinqiao Hospital, Third Military Medical University, Chongqing 400037, China

**Keywords:** cardiotoxicity, CHD9, ketamine, microRNA-208a, Notch, p65

## Abstract

**Background:** MicroRNA can regulate gene expression, and participate in multiple vital activities, such as inflammation, oxidative stress epigenetic modification, cell proliferation, and apoptosis. It plays an important role in the genesis and development of cardiovascular disease.

**Objective:** To assess the role of microRNA-208a in ketamine-induced cardiotoxicity.

**Methods:** All rats were randomly selected into two groups: sham and model groups. After fixed, all rats in the model group was intraperitoneally (IP) injected with 100 mg/kg of ketamine. Heart samples were stained with HE assay. Total RNAs from serum were used to hybridize with the SurePrint G3 Rat Whole Genome GE 8×60 K Microarray G4858A platform.

**Results:** In the rat model with ketamine-induced cardiotoxicity, microRNA-208a expression was increased. Then, over-expression of microRNA-208a increased inflammation and oxidative stress *in vitro* model. However, down-regulation of microRNA-208a decreased inflammation and oxidative stress *in vitro* model. Over-expression of microRNA-208a suppressed CHD9 and Notch1, and induced p65 protein expression *in vitro* model. Overexpression of CHD9 reduced the effects of microRNA-208a on inflammation and oxidative stress in heart cell through Notch/p65 signal pathways. Notch1 activation reduced the effects of microRNA-208a on inflammation and oxidative stress in heart cell through p65 signal pathways.

**Conclusion:** MicroRNA-208a may be a potential biomarker for ketamine-induced cardiotoxicity through inflammation and oxidative stress by Notch/NF-κB signal pathways by CHD9.

## Introduction

Ketamine is one of the classical general anesthetics, which has been applied in clinic for over 40 years. It can be mainly divided into racemic ketamine and dextral ketamine [1]. Of them, racemic ketamine is the racemate of two enantiomers of dextral ketamine and levorotatory ketamine. The two are partially different, but they both have little influence on the circulation [2]. Only the dextral ketamine has slightly enhanced suppression on the left ventricular systolic function. Research indicates that intravenous injection of ketamine can increase the cardiac index in normal patients by 40–50% [2]. Also, it is reported that ketamine application can reduce the blood pressure and periphery circulation resistance, and lower the cardiac index [3]. In recent years, ketamine has attracted attention due to its different effects on blood pressure [3].

In patients with cardiac toxicity, endomyocardial biopsy manifests as lymphocyte, neutrophil, and macrophage infiltration in heart, along with cardiac fibrosis [4]. Moreover, patients with stress cardiomyopathy at the acute stage show myocardial edema in heart MRI. Typically, edema is the major manifestation of acute myocardial inflammatory reaction [4]. Numerous subsequent cardiovascular examinations reveal the presence of inflammatory process. With the recovery of disease, the left ventricular function reveals that the T2-phase abnormality of inflammation can recover [5]. The inflammatory factors such as IL-17, MMP-9, and Caspase-3 were significantly higher in the cardiac muscle after chronic fluoride exposure [6]. These results indicated that inflammation or focal myocarditis may be one of the pathogenesis of stress cardiomyopathy [5].

NF-κB is the key regulatory factor of inflammatory response. The IκB kinase (IKK) is a mixture of multiple subunits, including catalytic subunits like IKK-α, β, and γ [7]. So far, two main NF-κB activation pathways based on ligand and surface receptor have been determined [8]. The canonical pathway relies on IKK-γ and IKK-β to induce inflammation and gene transcription for cell survival. Conversely, the non-canonical NF-κB activation is mainly regulated by the development of B cells [8]. The canonical pathway can be triggered by the infection factors and pro-inflammatory cytokines [7].

Oxidative stress is a severe tissue cell injury process resulting from excessive accumulation of reactive oxygen species (ROS) and reactive nitrogen species (RNS) under the stimulation of various physicochemical factors [9]. NADPH oxidase (Nox) is one of the major sources of ROS and RNS in myocardial tissues [10]. The current study verifies that Nox participates in regulating multiple cardiovascular diseases, including cardiac failure, atherosclerosis, myocardial ischemia reperfusion injury (MI/RI), hypertension, and cardiomyopathy [11]. In the MI/RI model, Nox2 and Nox4 expression is markedly up-regulated, and the ROS levels are also elevated. This finding reveals that Nox plays a vital role in regulating MI/RI [11].

The Notch signal transduction pathway was first observed by Otto L. Mohr in drosophila melanogaster in 1900, while the *Notch* gene was first cloned in 1983 [12]. Notch can regulate cell differentiation, apoptosis, proliferation, and morphogenesis [12]. Besides, it plays a crucial role in cell growth and development [12]. The Notch-mediated signal transmission plays a key role in cardiovascular development, as well as the genesis and development of cardiovascular diseases [13].

MicroRNA-208a is essential for the expression of the genes involved in cardiac hypertrophy and fibrosis [14]. MicroRNA-208a silencing could attenuate doxorubicin-induced myocyte apoptosis and cardiac dysfunction [15]. Plasma microRNA-208a could be used as a useful biomarker for drug-induced cardiotoxicity in rats [16]. However, the role of microRNA-208a and its biological mechanism are still unknown. So in the present study, the role of microRNA-208a in ketamine-induced cardiotoxicity and its effect on inflammation and oxidative stress were explored.

## Materials and methods

### Ketamine-induced animal models

A total of 20 Sprague–Dawley rats were obtained from Medicine Laboratory of Shenzhen University. All procedures were reviewed and approved by Shenzhen University General Hospital (Approval number or code: TMMU-371-02-01). All the rats were randomly selected into two groups: sham and model groups. After being fixed, all rats in the model group were intraperitoneally (IP) injected with 100 mg/kg of ketamine.

### Cell lines and cell culture

H9c2 cells were cultured at DMEM (Invitrogen; Carlsbad, CA, U.S.A.) supplemented with 10% fetal bovine serum (Invitrogen; Carlsbad, CA, U.S.A.) at 37˚C in 5% CO_2_. MicroRNA-208a, si-microRNA-208a, and negative control mimics were transfected into cell using Lipofectamine 2000 reagent (Invitrogen). After transfection for 48 h, H9c2 cells were achieved by 0.8 M of ketamine for 4 h.

### MiRNA microarray analysis

Total RNAs from serum were hybridized with the SurePrint G3 Rat Whole Genome GE 8×60 K Microarray G4858A platform (Stratagene). Images were quantified and analyzed using Agilent Feature Extraction Software (A.10.7.3.1).

### ELISA assay

ROS levels were measured using ROS assay and observed using Olympus BX51 microscope (Olympus BX51, CCD: DP71, Japan). Cells were collected at 1000 g for 10 min at 4°C and extracted using RIPA assay. SOD, GSH, GSH-PX, MDA, TNF-α, IL-1β, IL-6, and IL-18 levels were measured using ELISA kits.

### Luciferase reporter gene assays

The 3′-UTRs of CHD9 were subcloned into the psiCHECKTM-2 reporter vector (Promega, Madison, WI). The 3′-UTR CHD9 and microRNA-208a were co-transfected using Lipofectamine 2000 reagent (Invitrogen). The luciferase activity was measured at 48 h after transfection using a Dual-Luciferase Reporter Assay System (Promega).

### Histology assay

Heart samples were washed with PBS and fixed with 10% formalin for 48 h. Heart samples were then embedded in paraffin, and cut into 4 mm thick sections. Thick sections were stained with HE assay for 5 min.

### Reverse transcription-quantitative PCR (qPCR)

Total RNAs from serum and cells were isolated using Trizol reagent (Invitrogen, U.S.A.) according to the manufacturer’s instructions. Total RNAs were reversed to cDNA by Taqman MicroRNA Reverse Transcription kit (ThermoFisher Scientific, U.S.A.). The quantitative PCR (qPCR) analysis was carried out using ABI PRISM 7500 Real-time PCR System (Applied Biosystems, Foster City, CA) by SYBR Green Master Mix (Applied Biosystems) following thermal cycling profile: 95°C for 5 min, 40 cycles of amplification (95°C for 20 s, 60°C for 20 s, and 72°C for 20 s), and melt curve analysis. Sequences of U6: CTCGCTTCGGCAGCACA and AACGCTTCACGAATTTGCGT.

### Western blot analysis

Cellular proteins were extracted in RIPA buffer and protein concentration was quantified by Bradford assay. Proteins were separated by 10% SDS–PAGE and transferred to polyvinylidene difluoride (PVDF) membranes. Membranes were blocked in 5% fat-free milk dissolved in TBST and incubated with CHD9, Notch, p65, and GAPDH (Santa Cruz Biotechnology, Inc.) at 4°C over-expression. The membranes were then washed several times with 5 min and incubated with horseradish peroxidase-conjugated secondary antibody (Santa Cruz Biotechnology, Inc.) for 1 h at 37 °C. The membranes were detected with an ECL system (Millipore, Bedford, MA, U.S.A.) and quantified by Image Lab 3.0 (Bio-Rad Laboratories, Inc.).

### Statistical analysis

All the data were expressed as mean ± S.E.M. Statistical significance was assessed by comparing mean values by Student’s *t* test. A *P*-value of <0.05 was considered statistically significant.

## Results

### MicroRNA-208a expression in rat model of ketamine-induced cardiotoxicity

**Figure 1:**
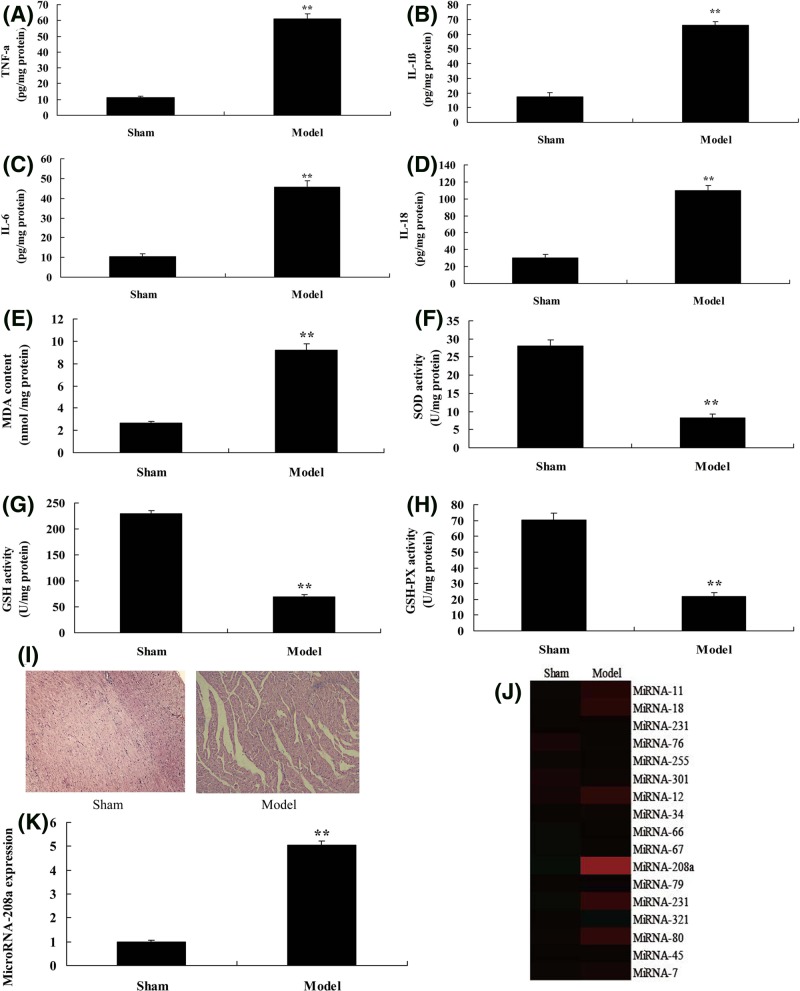
MicroRNA-208a expression in rat model of ketamine-induced cardiotoxicity TNF-α (**A**), IL-1β (**B**), IL-6 (**C**), IL-18 (**D**), MDA (**E**), and SOD (**F**); GSH (**G**), GSH-px (**H**) levels, and HE staining (**I**); heat map and qPCR for microRNA-208a expression (**J,K**). ^**^P<0.01 versus sham control group. Abbreviations: Model, ketamine-induced cardiotoxicity group; Sham, sham control group.

To explore the mechanism of microRNA-regulated ketamine-induced cardiotoxicity, the changes of microRNA in the rat model with ketamine-induced cardiotoxicity were analyzed. As shown in Figure 1A–D, the TNF-α, IL-1β, IL-6, and IL-18 levels were increased in heart tissue of ketamine-induced rat model, compared with sham group. SOD, GSH, and GSH-PX levels were increased, and MDA level was reduced in heart tissue of the model group, compared with sham group (Figure 1E–H). HE staining showed that myocardial fibrosis appeared in the model group, compared with sham group (Figure 1I). Then, microRNA-208a expression was increased (about 5-fold changes) in the model group, compared with sham group (Figure 1J,K). These results showed that MicroRNA-208a was kept in the rat model with ketamine-induced cardiotoxicity.

### MicroRNA-208a regulates Notch/p65 signal pathways *in vitro* model by CHD9

**Figure 2:**
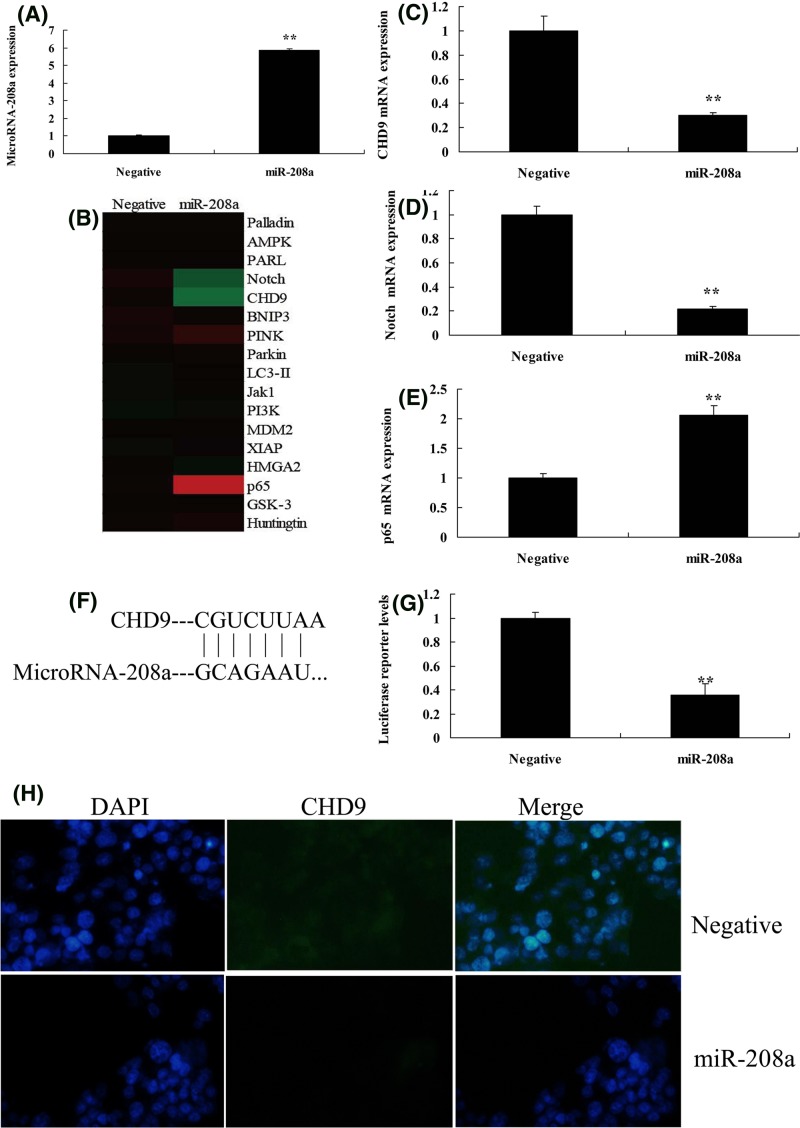
MicroRNA-208a regulates Notch/p65 signal pathways *in vitro* model by CHD9 The qPCR for microRNA-208a expression (**A**), hear map (**B**), CHD9 (**C**), Notch1 (**D**), and p65 (**E**) expression; 3’UTR region of CHD9 showed potential alignment with microRNA-208a sequence (**F**); luciferase reporter activity levels (**G**), IF for CHD9 protein expression (**H**). ^**^P<0.01 versus negative control group. Abbreviations: miR-208a, over-expression of microRNA-208a group; Negative, negative control group.

**Figure 3:**
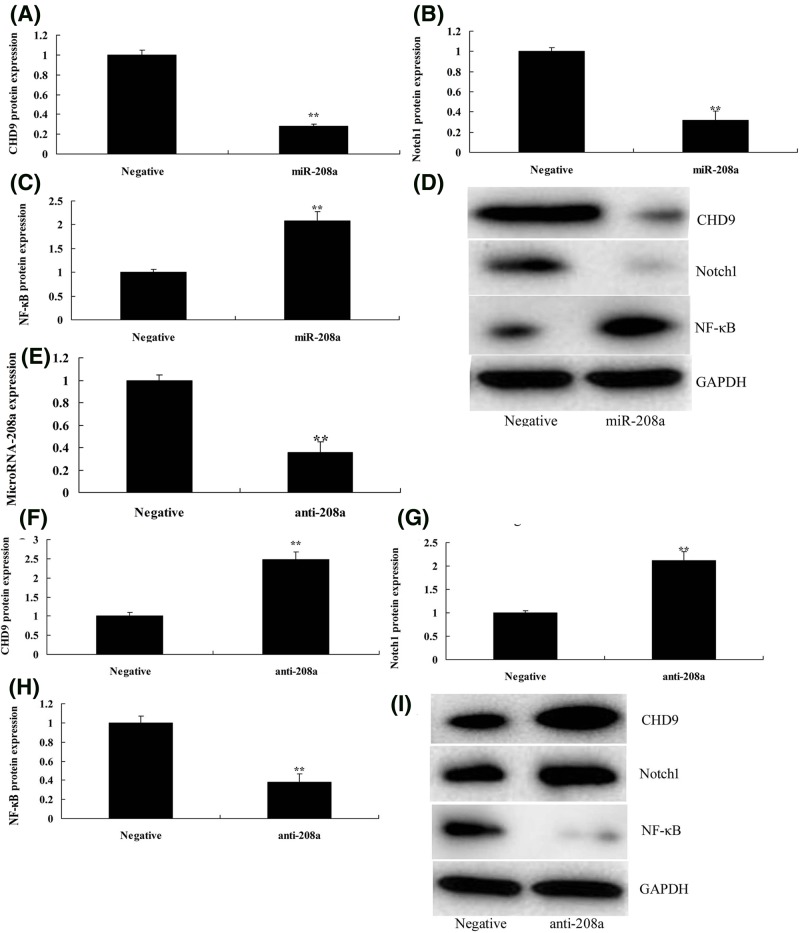
MicroRNA-208a regulates CHD9/Notch/p65 protein expression *in vitro* model CHD9, Notch, and p65 protein expression (**A–C**) by Western blotting; CHD9, Notch, and p65 protein expression (**D**) by statistical analysis using over-expression of microRNA-208a; qPCR for microRNA-208a expression (**E**); CHD9, Notch, and p65 protein expression (**F**–**H**) by Western blotting; CHD9, Notch, and p65 protein expression (**I**) by statistical analysis using down-regulation of microRNA-208a. ^**^P<0.01 versus negative control group. Abbreviations: anti-208a, down-regulation of microRNA-208a group; miR-208a, over-expression of microRNA-208a group; Negative, negative control group.

Then, the function of microRNA-208a was analyzed in ketamine-induced *vitro* model. As showed in Figure 2A, microRNA-208a mimics were transfected into ketamine-induced *vitro* model, and the expression of microRNA-208a was increased. Gene chip was used to analyze the changes of signal pathways *in vitro* model by the over-expression of microRNA-208a. As shown in Figure 2B–E, the over-expression of microRNA-208a suppressed CHD9 and Notch1, and induced p65 expression *in vitro* model, compared with negative group. Figure 2F,G showed that 3’UTR region of CHD9 showed potential alignment with microRNA-208a sequence and luciferase reporter activity levels were reduced by over-expression of microRNA-208a, compared with negative group. Over-expression of microRNA-208a suppressed the protein expression of CHD9 *in vitro* model, compared with negative control group (Figure 2H). Then, over-expression of microRNA-208a suppressed CHD9 and Notch1, and induced p65 protein expression *in vitro* model, compared with negative control group (Figure 3A–D). However, anti-microRNA-208a mimics reduced the expression of microRNA-208a *in vitro* model, compared with negative group (Figure 3E). Down-regulation of microRNA-208a induced CHD9 and Notch1, and suppressed p65 protein expression *in vitro* model, compared with negative control group (Figure 3F–I). So, these results showed that microRNA-208a could regulate Notch/p65 signal pathways *in vitro* model by CHD9.

### MicroRNA-208a regulates inflammation *in vitro* model by CHD9

**Figure 4:**
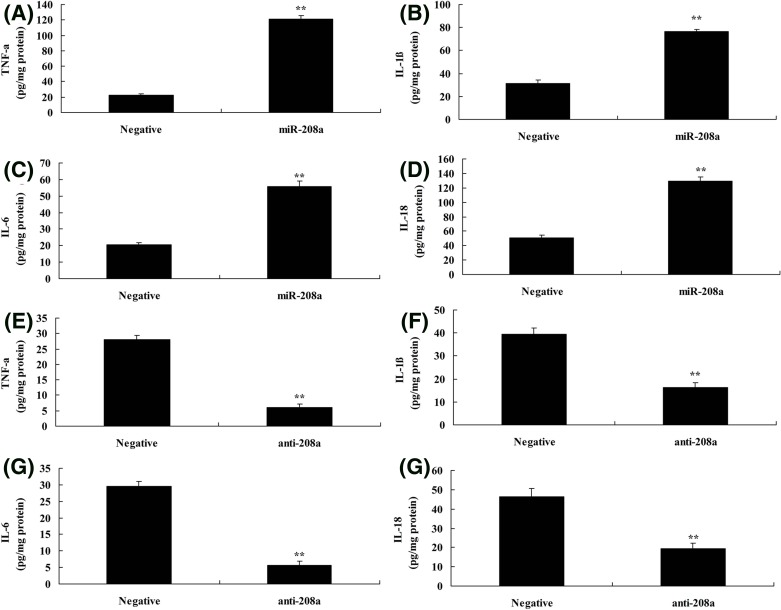
MicroRNA-208a regulates inflammation *in vitro* model by CHD9 TNF-α (**A**), IL-1β (**B**), IL-6 (**C**), IL-18 (**D**) using over-expression of microRNA-208a; TNF-α (**E**), IL-1β (**F**), IL-6 (**G**), IL-18 (**H**) using down-regulation of microRNA-208a. ^**^P<0.01 versus negative control group. Abbreviations: anti-208a, down-regulation of microRNA-208a group; miR-208a, over-expression of microRNA-208a group; Negative, negative control group.

Next, microRNA-208a regulates p65 signal pathways, so we analyzed the effects of microRNA-208a on inflammation *in vitro* model. As shown in Figure 4A–D, the over-expression of microRNA-208a increased TNF-α, IL-1β, IL-6, and IL-18 levels *in vitro* model, compared with negative group. Down-regulation of microRNA-208a reduced TNF-α, IL-1β, IL-6, and IL-18 levels *in vitro* model, compared with negative group (Figure 4E–H). The present study showed that microRNA-208a regulated inflammation *in vitro* model by CHD9/Notch/p65 signal pathways.

### MicroRNA-208a regulates oxidative stress *in vitro* model by CHD9

**Figure 5:**
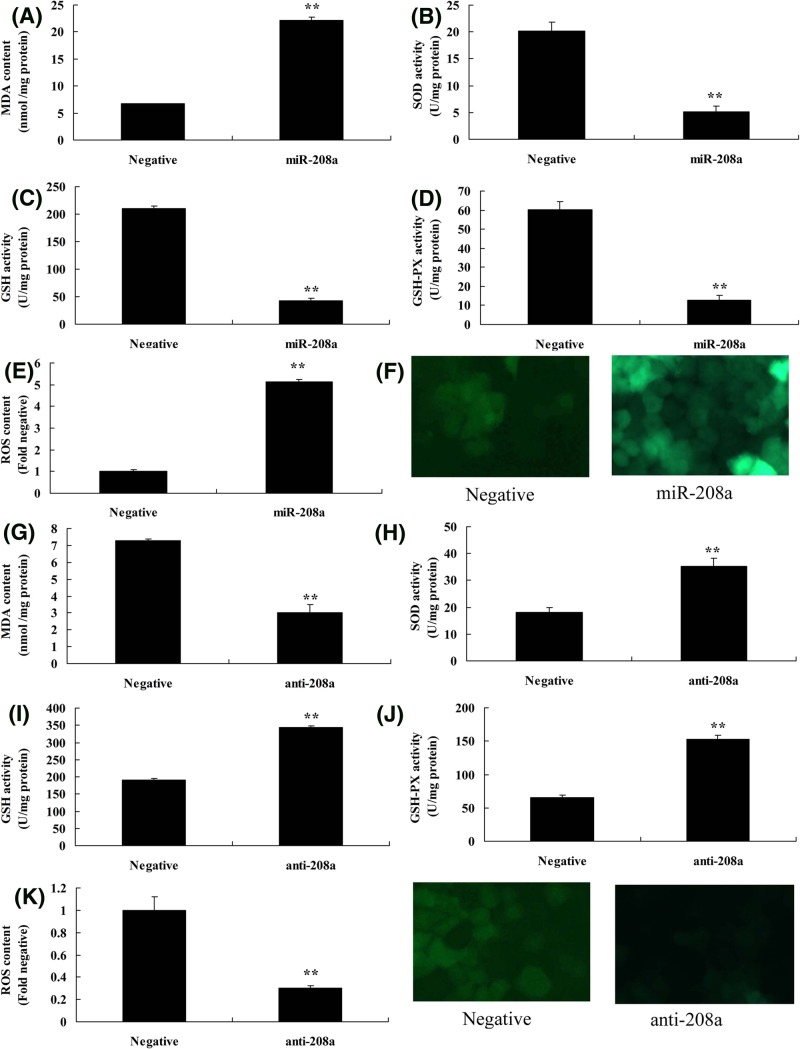
MicroRNA-208a regulates oxidative stress *in vitro* model by CHD9 MDA (**A**), SOD (**B**), GSH (**C**), GSH-px (**D**) levels, and ROS levels (**E,F**) using over-expression of microRNA-208a; MDA (**G**), SOD (**H**), GSH (**I**), GSH-px (**J**) levels, and ROS levels (**K,L**) using down-regulation of microRNA-208a. ^**^P<0.01 versus negative control group. Abbreviations: anti-208a, down-regulation of microRNA-208a group; miR-208a, over-expression of microRNA-208a group; Negative, negative control group

Then it was analyzed whether microRNA-208a played a role in oxidative stress *in vitro* model. Figure 5A–F, showed that over-expression of microRNA-208a promoted MDA levels and ROS content, and inhibited SOD, GSH, and GSH-px levels *in vitro* model, compared with negative group. Moreover, down-regulation of microRNA-208a reduced MDA levels and ROS content, and increased SOD, GSH, and GSH-px levels *in vitro* model, compared with negative group (Figure 5G–L). Thus, microRNA-208a also regulated oxidative stress to reduce ROS levels *in vitro* model by CHD9/Notch signal pathways.

### Si-CHD9 reduced the effect of microRNA-208a on oxidative stress and inflammation *in vitro* model

**Figure 6:**
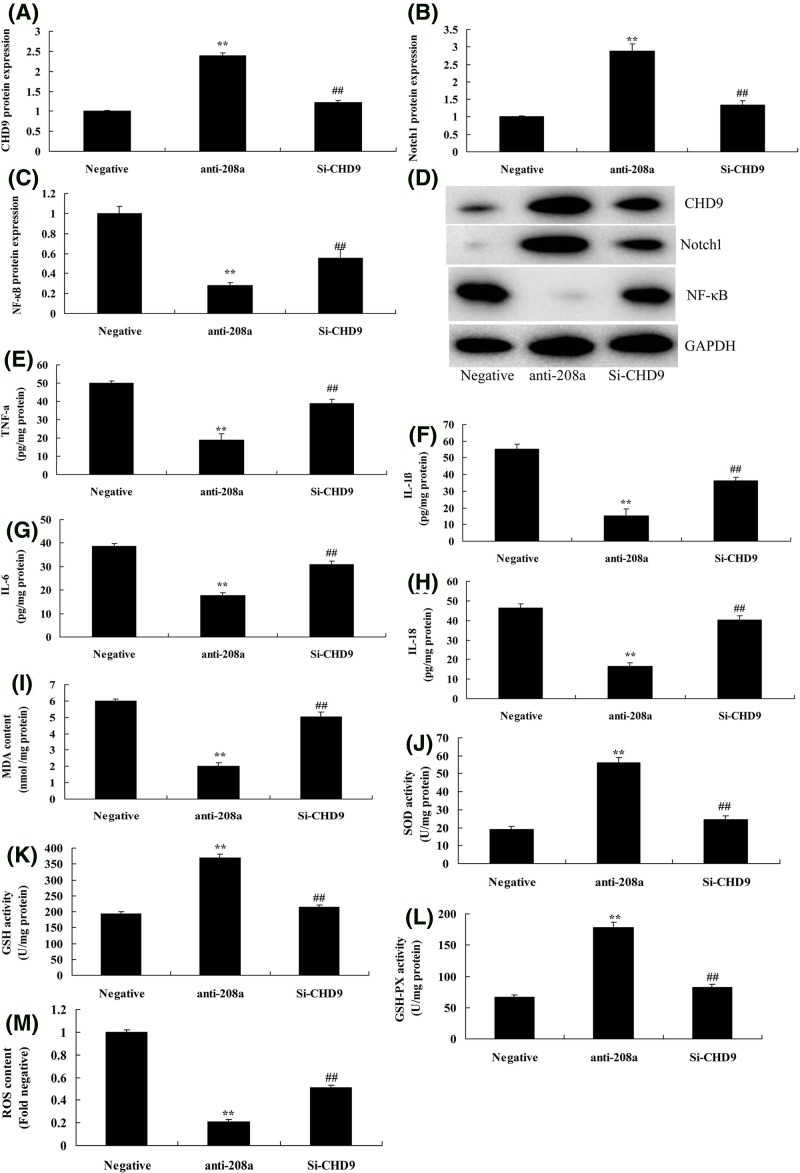
Si-CHD9 reduced the effect of microRNA-208a on oxidative stress and inflammation *in vitro* model CHD9, Notch, and p65 protein expression (**A**–**C**) by Western blotting; CHD9, Notch, and p65 protein expression (**D**) by statistical analysis; TNF-α (**E**), IL-1β (**F**), IL-6 (**G**), IL-18 (**H**), MDA (**I**), SOD (**J**), GSH (**K**), GSH-px (**L**) levels, and ROS levels (**M**). ^**^P<0.01 versus negative control group; ^##^*P*<0.01 versus down-regulation of microRNA-208a group. Abbreviations: anti-208a, down-regulation of microRNA-208a group; Negative, negative control group; Si-CHD9, Si-CHD9 and down-regulation of microRNA-208a group.

The role of CHD9 in the effect of microRNA-208a on oxidative stress and inflammation *in vitro* model was further explored. As shown in Figure 6A–D, si-CHD9 suppressed CHD9 and Notch1, and induced p65 protein expression *in vitro* model by down-regulation of microRNA-208a, compared with down-regulation of microRNA-208a group. Then, results found that Si-CHD9 reduced the effect of microRNA-208a on the activation of MDA, ROS content, TNF-α, IL-1β, IL-6, and IL-18 levels, and inactivation of SOD, GSH, and GSH-px levels *in vitro* model, compared with down-regulation of microRNA-208a group (Figure 6E–M).

### Si-Notch reduced the effect of microRNA-208a on oxidative stress and inflammation *in vitro* model

**Figure 7:**
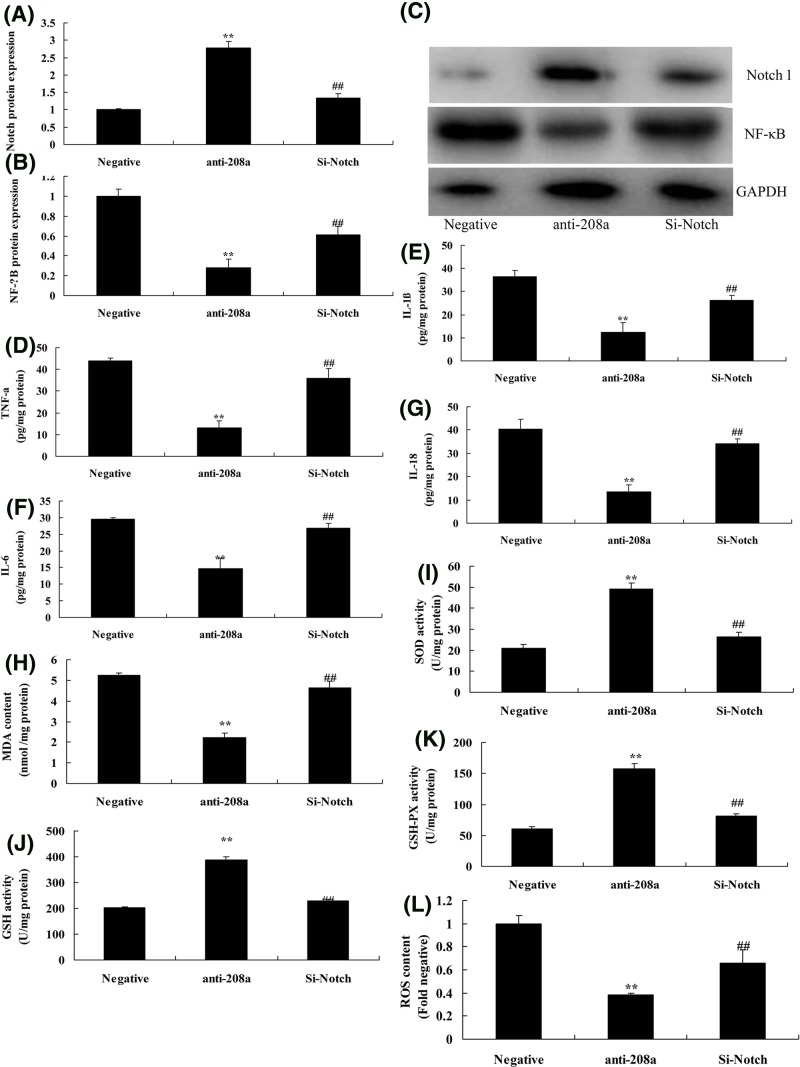
Si-Notch reduced the effect of microRNA-208a on oxidative stress and inflammation *in vitro* model Notch and p65 protein expression (**A,B**) by Western blotting; Notch and p65 protein expression (**C**) by statistical analysis; TNF-α (**D**), IL-1β (**E**), IL-6 (**F**), IL-18 (**G**), MDA (**H**), SOD (**I**), GSH (**J**), GSH-px (**K**) levels, and ROS levels (**L**). ^**^P<0.01 versus negative control group; ^##^*P*<0.01 versus down-regulation of microRNA-208a group. Abbreviations: anti-208a, down-regulation of microRNA-208a group; Negative, negative control group; Si- Notch, Si- Notch and down-regulation of microRNA-208a group.

To analyze the effect of microRNA-208a on oxidative stress and inflammation in vitro model, the expression of Notch protein was suppressed in si-Notch group, compared with anti-microRNA-208a group (Figure 7A,C). Then, Si-Notch also induced the protein expression of p65, and increased TNF-α, IL-1β, IL-6, and IL-18 levels, and promoted MDA levels and ROS content, and reduced SOD, GSH, and GSH-px levels *in vitro* model by down-regulation of microRNA-208a, compared with only down-regulation of microRNA-208a group (Figure 7B–L).

### Si-p65 reduced the effect of microRNA-208a on inflammation *in vitro* model

**Figure 8:**
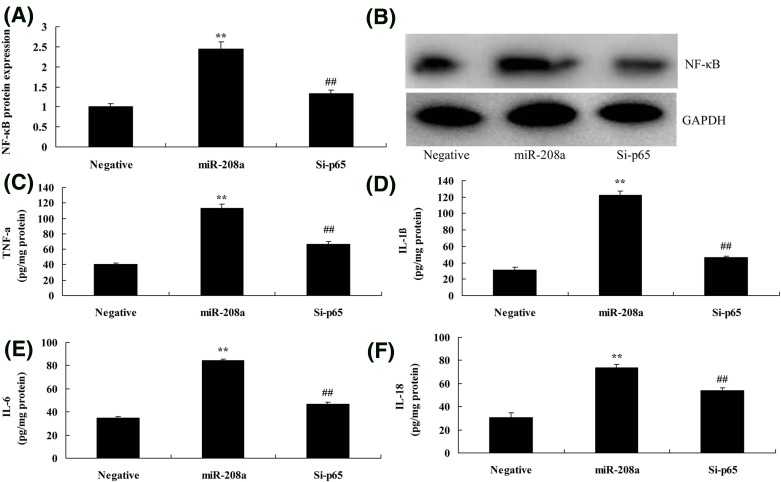
Si-p65 reduced the effect of microRNA-208a on inflammation *in vitro* model The p65 protein expression (**A**) by Western blotting; Notch and p65 protein expression (**B**) by statistical analysis; TNF-α (**C**), IL-1β (**D**), IL-6 (**E**), IL-18 (**F**). ^**^P<0.01 versus negative control group; ^##^*P*<0.01 versus down-regulation of microRNA-208a group. miR-208a, over-expression of microRNA-208a group; Negative, negative control group; Si-p65, Si-p65 and over-expression of microRNA-208a group.

To further examine the role of p65 in the effect of microRNA-208a on inflammation *in vitro* model, si-p65 reduced the protein expression *in vitro* model of down-regulation of microRNA-208a, compared with over-expression of microRNA-208a group (Figure 8A,B). Si-p65 reduced TNF-α, IL-1β, IL-6, and IL-18 levels *in vitro* model by down-regulation of microRNA-208a, compared with down-regulation of microRNA-208a group (Figure 8C–F).

## Discussion

Ketamine has different effects on angiotasis among different populations [2]. To be specific, it can elevate the systemic circulation and pulmonary circulation pressure in normal subjects, while decrease those in critically ill patients [17]. The ketamine-resulted blood pressure elevation is mainly related to the sympathetic nerve-induced release of catecholamine [17]. In contrast, the direct relaxant effect of ketamine on blood vessels will reduce the blood pressure. Ketamine can relax the systemic circulation and pulmonary circulation blood vessels *in vitro* [17,18]. Its mechanism is that ketamine can suppress the voltage-gated calcium channel, reduce calcium influx, and lower the intracellular calcium ion concentration [19]. Ketamine can suppress calcium ion release in the sarcoplasmic reticulum, and such effect varies among different species and blood vessels [19]. In the present study, microRNA-208a expression was increased in heart tissue of ketamine-induced rat model. Liu et al. [20] showed that microRNA-208a promoted apoptosis and oxidative stress by H_2_O_2_ in cardiomyocytes.

Inflammatory response is the endogenous stress response in the body after myocardial infarction. Appropriate inflammatory response early after myocardial infarction can protect the survived myocardial cells [9]. This is beneficial for myocardial healing after infarction, against myocardial dilation, and increasing the tensile strength of myocardial scar after infarction. However, excessive and persistent inflammatory response plays a vital part in the development of ventricular remodeling. Our results also suggested that microRNA-208a reduced inflammation and oxidative stress *in vitro* model. Wang et al. [21] showed that microRNA-208a was significantly related to IL-6 in coronary artery bypass grafting surgery.

Notch1 mainly expresses in the intima of the original cardiac efferent tract in early mouse embryo [22]. Therefore, it has important regulatory effect on the capacity of the original cardiovascular endometrial cells. The genesis of aortic valve disease can be induced by deletion mutation of Notch2 signal transduction function. This verifies the significance of Notch1 in the development of endometrial cells [22]. The defective Notch signal transduction in endocardium will affect the development of cardiac valves and ventricles. This will lead to a series of diseases, including bicuspid valve aortic valvuloplasty and non-compressible cardiomyopathy in left ventricle. Notch signal transduction is also involved in the formation of epicardium and coronary artery [11]. Notch as well as its downstream target genes and inflammatory effectors can be mediated by the activated macrophage. This process can promote the genesis of inflammation and induce the formation of atherosclerosis. Genetic or pharmacological suppression of Notch1 can reduce NF-κB phosphorylation and nuclear translocation to lessen plaque formation and inflammatory response. Macrophage development and the immune response induced by lipopolysaccharide (LPS) stimulation can also be regulated by Notch [19]. Such regulatory effect is achieved through regulating the expression of macrophage surface marker CD11b [23]. In the present study, we found that microRNA-208a regulated Notch/p65 signal pathways *in vitro* model by CHD9. Notch1 activation reduced the effects of microRNA-208a on cardiotoxicity in heart cell through p65 signal pathways. Chistiakov et al. [24] reported that miR-208 was involved in the late cardiogenic stages mediating differentiation of cardioblasts to cardiomyocytes. Zhang et al. [25] showed that microRNA-208a regulated H9c2 cells simulated ischemia-reperfusion myocardial injury via CHD9 through Notch/NF-κB signal pathways.

NF-κB is a pattern recognition receptor, which forms important association between congenital immunity and acquired immunity [26]. NF-κB is distributed in immune cells; meanwhile, it is also highly expressed in cardiovascular system, such as vascular endothelial cells and myocardial cells. A large number of studies indicate that, the NF-κB signaling pathway is closely correlated with the genesis and development of cardiovascular disease [8]. Current studies mainly focus on the pathogenesis of hypertension, atherosclerosis, viral myocarditis, and septicemia [9]. These tend to believe that there is a set of congenital immune system with complete function in the myocardium [9]. In the present study, we found that si-p65 reduced the effect of microRNA-208a on inflammation *in vitro* model. Zhang et al. [25] showed that microRNA-208a regulated H9c2 cells simulated ischemia-reperfusion myocardial injury via CHD9 through Notch/NF-κB signal pathways.

**Figure 9:**
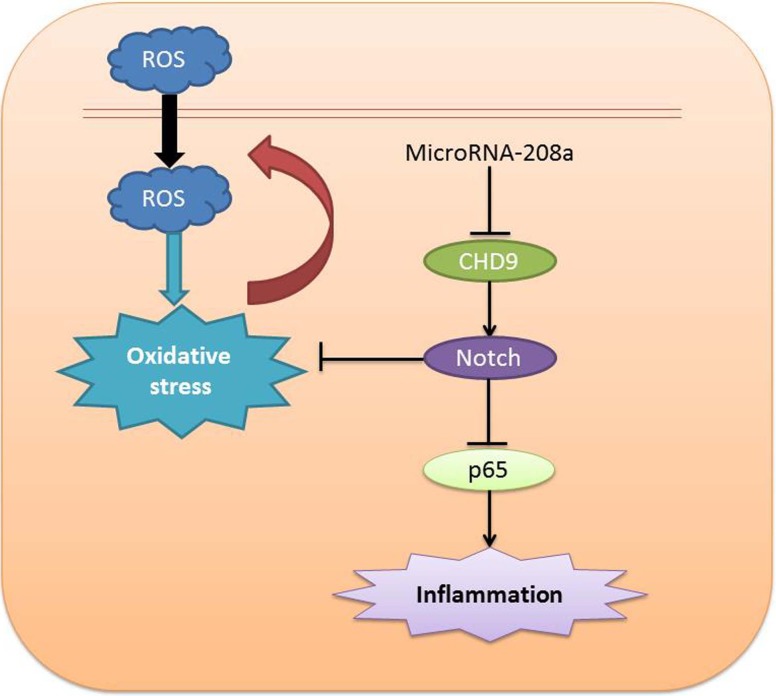
MicroRNA-208a as potential biomarkers for ketamine-induced cardiotoxicity through pro-inflammation by Notch/NF-κB signal pathways by CHD9

In conclusion, the present study showed that microRNA-208a reduced inflammation and oxidative stress *in vitro* model through Notch/NF-κB signal pathways by CHD9 (Figure 9). Moreover, microRNA-208a may be a potential biomarker for ketamine-induced cardiotoxicity. The further study *in vivo* and in animal models about microRNA-208a as a potential biomarker for clinical diagnosis will be helpful in the early diagnosis and post-injury treatment of ketamine-induced cardiotoxicity.

## Abbreviations

GSH: glutathione
GSH-Px: glutathione peroxidase
IKK: IκB kinase
IL-17: interleukin-17
MDA: methylenedioxyamphetamine
MI/RI: myocardial ischemia reperfusion injury
MMP-9: matrix metalloproteinase-9
Nox: NADPH oxidase
qPCR: quantitative PCR
RNS: reactive nitrogen species
ROS: reactive oxygen species
SOD: Superoxide dismutases
TNF-α: tumour-necrosis factor-α
